# P-2237. Mortality and Causative Pathogens of Sepsis Differ by Molecular Phenotype in a Secondary Analysis of MARS

**DOI:** 10.1093/ofid/ofae631.2390

**Published:** 2025-01-29

**Authors:** Brian Bartek, Manoj Maddali, Rombout B E van Amstel, Tom van der Poll, Olaf Cremer, Mars Consortium, Lieuwe D J Bos, Pratik Sinha, Carolyn S Calfee

**Affiliations:** Washington University in St. Louis, St. Louis, Missouri; Stanford University, Stanford, California; Amsterdam UMC location AMC, University of Amsterdam, Amsterdam, Noord-Holland, Netherlands; Amsterdam UMC location AMC, University of Amsterdam, Amsterdam, Noord-Holland, Netherlands; University Medical Center, Utrecht, Utrecht, Utrecht, Netherlands; Amsterdam University Medical Centers, University of Amsterdam, Mahidol University, University of Oxford and University Medical Centers Utrecht, Amsterdam, Noord-Holland, Netherlands; Amsterdam UMC location AMC, University of Amsterdam, Amsterdam, Noord-Holland, Netherlands; Washington University School of Medicine in St. Louis, St. Louis, MO; University of California San Francisco, San Francisco, California

## Abstract

**Background:**

Heterogeneity of host response has been a barrier to identifying novel therapies in sepsis. Two molecular phenotypes of critical illness, called the Hyperinflammatory and Hypoinflammatory, have been identified in multiple cohorts of sepsis and ARDS ( >12,000 patients), with divergent biology, clinical course, and responses to therapy^1^. We evaluated whether these phenotypes were identifiable in MARS-- a large observational cohort of critically ill patients-- and whether the pathogens present in each phenotype differed.

**Figure:**
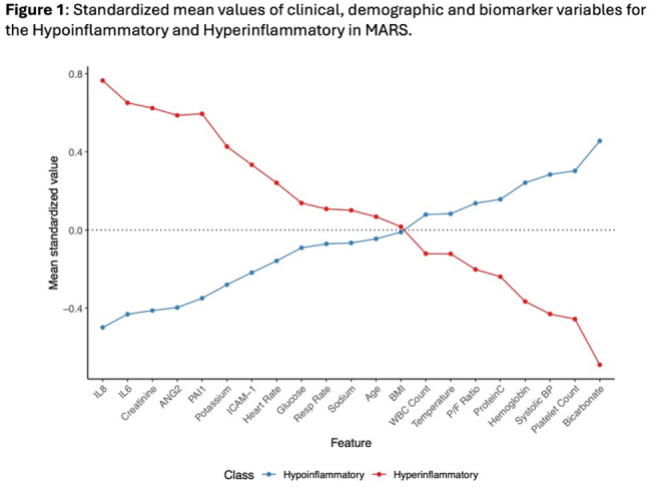

**Methods:**

MARS recruited critically ill patients from two Dutch ICUs. Only patients meeting sepsis or ARDS diagnostic criteria were included for this analysis. We applied latent class analysis (LCA) to vital signs, clinical laboratory values, and protein biomarkers from Day 1 of ICU admission in 1,485 patients. LCA models with 1-5 classes were fitted. We assigned class membership using the best fitting model and compared characteristics between classes. The present classes’ overlap with prior phenotypes was evaluated by comparison to an extensively validated parsimonious model,^2^ trained previously to classify molecular phenotypes.

**Results:**

A two-class model best fit the cohort (VLMR p< .001), and the parsimonious model and multivariate profiles suggested high overlap (AUC=.929) with previously described ‘Hypoinflammatory’ (Class 1; n=895) and ‘Hyperinflammatory’ (Class 2; n=590) phenotypes (Figure 1). Bacteremia was more prevalent in the Hyperinflammatory phenotype (27.2% vs 7.8%; p< .001), with Enterobacteriaceae as the most prevalent species. For primary infection sites, lower respiratory tract was more common in the Hypoinflammatory (56% vs 36%) and abdomen more common in the Hyperinflammatory phenotype (26% vs 9%). Mortality was significantly higher in the Hyperinflammatory phenotype at both 30 days (OR=2.54, CI_95%_= 2.003—3.235 ) and 90 days (OR=2.41, CI_95%_ = 1.925—3.016; both p< .001).

**Conclusion:**

In MARS, we identified the previously described Hypo- and Hyperinflammatory phenotypes with divergent host response, pathogen profiles, and outcomes. These findings suggest that host phenotype signatures in part may be driven by sites of infection and pathogen species.

**Disclosures:**

Lieuwe D.J. Bos, MD-PhD, Astra Zeneca: Advisor/Consultant|CSL Behring: Advisor/Consultant|Impentri: Advisor/Consultant|Novartis: Advisor/Consultant|Scailyte: Advisor/Consultant|Sobi: Advisor/Consultant Pratik Sinha, MBChB/PhD, AstraZeneca: Advisor/Consultant|Prenosis Inc: Board Member Carolyn S. Calfee, MD, Cellenkos: Advisor/Consultant|Gen1e Life Sciences: Advisor/Consultant|Janssen: Advisor/Consultant|NGMBio: Advisor/Consultant|Santhera: Advisor/Consultant|Vasomune: Advisor/Consultant

